# How Healthy Are Community Health Workers: Findings from a Chicago-Area Survey

**DOI:** 10.1007/s10900-026-01621-7

**Published:** 2026-08-10

**Authors:** Melissa Gutierrez-Kapheim, Patricia Labellarte, Meghan R. Fortune, Adlaide Holloway, Jessica Bishop-Royse, Helen Margellos-Anast, Molly Martin

**Affiliations:** 1 Sinai Urban Health Institute, Sinai Chicago, 1500 South Fairfield Avenue, Room K438, Chicago, IL 60608 USA; 2 School of Public Health, University of Illinois Chicago, Chicago, IL USA; 3 Institute for Health Research and Policy, University of Illinois Chicago, Chicago, IL USA; 4 Department of Adult Health and Gerontological Nursing, Rush University College of Nursing, Chicago, IL USA

**Keywords:** Community health worker, Occupational health, Survey research, Chronic disease, Mental health

## Abstract

Community health workers (CHWs) are a growing and essential component of the U.S. healthcare workforce, yet little is known about their own health and well-being. The CHWs Our Work + Our Health Pilot Study administered a cross-sectional survey to a convenience sample of CHWs living and/or working in the Chicago metropolitan area. The survey assessed general health, chronic conditions, health behaviors, and mental health using validated, standardized measures. Prevalence estimates were compared to Chicago population data using two-proportion z-tests. The final analytic sample included 179 CHWs, predominantly female (85.5%), Hispanic (63.7%) or non-Hispanic Black (32.4%). CHWs reported significantly worse outcomes than the general Chicago population in general health, mental distress, emotional support, and sleep, while reporting lower rates of hypertension, obesity, and high cholesterol. Nearly one-third reported fair or poor health and nearly 40% reported inadequate emotional support. Economic hardship was substantial, with 35.9% earning below $25,000 annually and 61.9% delaying medical care due to cost. Chicago-area CHWs present a mixed but concerning health profile marked by significant mental health burden and economic precarity, underscoring the need for targeted employer and policy investments to sustain this essential workforce.

## Introduction

Community health workers (CHWs) are frontline health workers who serve as trusted connectors between communities and health and social services [1]. Their shared experiences, cultural concordance, and authentic relationships uniquely position CHWs to reach individuals and communities facing complex health and social challenges [2–5]. CHWs provide a range of supports to their clients and communities including health education, informal counseling, social support, and advocacy. Their integration into healthcare delivery has demonstrated positive outcomes across many conditions [6]. As a result, the U.S. CHW workforce is growing, and most states are developing CHW certification standards and reimbursement models [7].

Despite their critical role in supporting health, little is known about the health and wellbeing of CHWs themselves. Research focused on the occupational health of the broader healthcare workforce has documented elevated risks for cardiovascular disease and mental wellbeing [8, 9]. Yet CHWs have been largely excluded from this body of research.

Filling this gap in knowledge about the well-being of the CHW workforce is especially important given the nature of CHW work. CHWs frequently work in community settings marked by high levels of disinvestment, and they often live in the same communities they serve thus they are directly experiencing the same challenges as their clients, including poverty, chronic illness, and exposure to violence [10]. The median annual CHW salary was $48,200 in 2024 [11], well below the U.S. median [12], raising further questions about cumulative strain. Without a clear picture of CHW health, employers and policymakers cannot prevent burnout, reduce turnover, and sustain this growing workforce.

To address this gap, the CHWs Our Work + Our Health Pilot Study was conducted among Chicagoland area CHWs. This paper describes the study design, data collection process, and prevalence of chronic health conditions of the sample and compares them to the Chicago population.

## Methods

### Study Design

This pilot study is part of the Chicagoland Community Engagement Alliance (CEAL) Program, a partnership of academic and community health experts that addresses health and social outcomes by increasing engagement in research and access to resources in the Chicago metro area and is a regional team in the NIH-funded National CEAL Program (OT2HL161610, SUB-OTA No. 6922-03-COVID-S020). This study was organized into three phases. Phase 1 consisted of a cross-sectional survey administered to a convenience sample of CHWs living and/or working in the Chicago metropolitan area. Phase 2 consisted of interviews with a subset of Phase 1 respondents, designed to contextualize interpretation of survey findings. Phase 3 consisted of a one-year follow-up survey administered to the Phase 1 cohort, generating longitudinal data on changes in CHW health and work experiences. This manuscript reports Phase 1 cross-sectional findings only. This study was approved by the Chicago Area Institutional Review Board (#2025 − 0891).

### Community-Engaged Approach

The study uses a community-engaged approach throughout all phases of design and implementation. The research team collaborates with three advisory bodies: the Chicagoland CEAL Community Advisory Board, the Illinois CHW Network, and a group of CHWs recruited specifically for their input on the study. These groups reviewed all aspects of survey design and implementation to ensure relevance to the lived and work experiences of Chicagoland CHWs.

Input was gathered on the survey instrument and recruitment strategies through four structured sessions: two with CHWs and two with the Chicagoland CEAL Community Advisory Board. The research team, which included a CHW researcher, synthesized feedback from both sets of sessions and revised the survey accordingly, aligning CHW-identified priorities with standardized, validated measures to enable meaningful comparisons across populations.

### Study Population

To be eligible, participants were required to: (1) identify as a CHW; (2) be 18 years of age or older; (3) have worked in a CHW role for at least one year; (4) be able to communicate in English or Spanish; and (5) and live or work in the Chicagoland area. Because CHWs are referred to by a variety of job titles, eligibility was determined using a dual criterion approach based on both job title and job duties, consistent with established CHW workforce research [13]. Eligible titles included but were not limited to community health worker, promotora de salud, patient navigator, health advisor, peer educator, outreach worker, and patient educator [14]. Eligible job duties included community engagement and advocacy, navigation and coordination of services, health education, serving as a community or cultural liaison, among others [14].

### Measures

The survey assessed six broad domains: (1) Demographics characteristics, (2) general health, (3) health risk behaviors, (4) mental health, (5) social and economic factors, and (6) employment and job satisfaction (Table 1). This manuscript reports on measures from the first four domains.

**Table 1:** Survey domains, questions, and sources

Domain	Details	Source
Demographics	Home zip code, age, sex, gender, race/ethnicity, languages spoken at home other than English, highest degree, number of people in household, household income	
General health	Overall general health	NIOSH WellBQ^66^
	Medical diagnoses, associated treatments	BRFSS and National CEAL
	Health-related work limitations and injuries	NIOSH WellBQ^66^
Health risks	Days per week engaged in moderate to vigorous physical activity (MVPA)	Sallis et al.^67^
	Minutes per week engaged in MVPA	Modified from Sallis et al.^67^
	Fruit and vegetable intake	BRFSS
	Sugar-sweetened beverage intake	BRFSS
	Water intake	
	Sleep per night	NIOSH WellBQ^66^
	Tobacco use	BRFSS and NIDA TAPS Tool^68^
	Alcohol use	NIDA TAPS Tool^68^
	Marijuana use	NIDA TAPS Tool^68^
Mental health	General well-being (standard of living, your health, what you are achieving, personal relationships, safety, part of community)	International Wellbeing Group^69^
	Emotional and social support	BRFSS
	Activity engagement	NIOSH WellBQ^66^
	Mental distress	BRFSS
	Depression/anxiety	PHQ-2, GAD-2^70-72^
	Isolation	Hughes et al.^73^
	Trauma	PC-PTSD^74^
	Superwoman Schema Scale	Woods-Giscombe^75^
Social and economic factors	Type of health insurance	National CEAL
	Place where participant gets medical care most often	National CEAL
	Had to wait for medical care because of cost	NHIS
	See people physically active in neighborhood	PANES^76^
	Own or rent housing	CDC BRFSS 2015^77^
	Housing challenges (pests, mold, etc.)	AHC HRSN^78^
	Worry about paying rent/mortgage	CDC BRFSS 2015^77^
	Had to cut size of meals	USDA Food Security^79^
	Have money left over at end of month	CFPB Financial Scale^80^
	Experiences of discrimination	NIOSH WellBQ^66^
	Trusted sources of health information	National CEAL
Employment	Current employment status	NIOSH WellBQ^66^
	Job security	NIOSH WellBQ^66^
Job satisfaction	Job satisfaction	NIOSH WellBQ^66^
	Supervisor and coworker support	NIOSH WellBQ^66^
	Job autonomy	NIOSH WellBQ^66^
	Time paucity/work overload	NIOSH WellBQ^66^
	Burnout	MiniZ^82^
	Control over workload, time for work	MiniZ^82^
	Work meaningfulness	NIOSH WellBQ^66^
	Supportive work culture	NIOSH WellBQ^66^
	Management trust	NIOSH WellBQ^66^
	Health culture at work	NIOSH WellBQ^66^
	Personal and work life balance	NIOSH WellBQ^66^
	Workplace safety	NIOSH WellBQ^66^
	Workplace/schedule flexibility	NIOSH WellBQ^66^
	Work-related fatigue	NIOSH WellBQ^66^

General health was assessed using items from the NIOSH WellBQ [15], including self-rated general health and health-related work limitations. Self-reported medical diagnoses were collected using items adapted from the BRFSS [16].

Health risk behaviors were assessed across seven domains. Physical activity was measured using one item adapted from Sallis et al. [17]: days per week engaged in moderate to vigorous physical activity. Fruit and vegetable intake and sugar-sweetened beverage consumption were assessed using BRFSS items [16]. Sleep duration was assessed using the NIOSH WellBQ [15]. Tobacco, alcohol, and marijuana use were assessed using BRFSS items and the NIDA TAPS [18].

Mental health and well-being were assessed across six domains. Emotional and social support and mental distress over the past 30 days were assessed using BRFSS items. Depression and anxiety symptoms were screened using the PHQ-2 and GAD-2, respectively [19, 20]. Social isolation was assessed using one question from the Hughes et al. loneliness measure [21]. Trauma exposure was assessed using the PC- PTSD-5 [22]. The Superwoman Schema Scale was included to assess culturally specific stress and coping patterns [23].

In addition to the health-focused measures, the survey assessed employment and job satisfaction domains. They captured current employment status and job security, as well as a range of workplace factors drawn primarily from the NIOSH WellBQ [15] including job satisfaction, supervisor and coworker support, job autonomy, work meaningfulness, management trust, supportive work culture, workplace safety, schedule flexibility, work-related fatigue, and personal and work-life balance. Burnout and control over workload were assessed using the Mini-Z instrument [24].

The social and economic factors domain assessed healthcare access and coverage, neighborhood environment, housing stability, financial strain, food security, and experiences of discrimination.

### Data Collection

Two participant-facing instruments were built in REDCap [25]: a brief eligibility screener and the full survey, both in English and Spanish. A recruitment email with was distributed to CHWs through a listserv maintained by the research team and through a network of partner community-based organizations. Additionally, a recruitment flyer with a QR code linking to the screener was distributed in person at a CHW conference.

Three eligibility questions assessed whether the respondent was 18 or older, identified as a CHW, and had worked as a CHW for at least one year. Respondents who answered “no” to any question were immediately notified of ineligibility. A “not sure” option was available for screening questions; respondents who selected this were offered a call with a study team member to determine eligibility. Eligible respondents were directed to download the study information sheet and were asked their preferred mode of survey administration (online or phone), along with their contact information.

The study team reviewed each submitted screener for eligibility and screened for duplicate or potentially fraudulent responses, including mismatched name and email combinations and suspiciously clustered submission, before issuing a survey invitation. Eligible respondents received a unique survey link via email, along with a link to the study information sheet. An automated reminder was sent one week after the initial invitation, followed by two additional email reminders sent manually through REDCap [25] and Outlook to reduce the likelihood of spam filtering. Respondents who requested phone administration were contacted by a team member. Participants received a $40 Tango digital gift card upon survey completion, distributed within one business day.

### Data Analysis

There are 179 survey respondents in the analytic sample. Participant characteristics are described using frequencies for categorical variables and mean with standard deviation for continuous variables. The prevalence of self-reported chronic health conditions, mental wellbeing, and behavioral risk factors in the CHW sample was compared to population estimates for the City of Chicago using two-proportion z-tests, with statistical significance set at α = 0.05. Chicago comparison data is drawn from the CDC Places Behavior Risk Factor Surveillance System 2022 [26] Chicago data. All analyses were conducted using Stata version 18 [27].

## Results

### Survey Recruitment and Response

Recruitment emails were distributed to 1,283 email addresses on a local CHW listserv; 189 (15%) were undeliverable. Because the listserv contained some individuals with multiple email addresses, the total number of unique individuals contacted was lower. A second recruitment email was sent to a randomly selected subset of 600 addresses from the original list. In addition, recruitment emails were sent to 30 community advisory board members and leaders at partner community-based organizations, who were asked to forward the invitation to CHWs in their networks. The screener was also distributed via flyer at a CHW conference; the number of flyers distributed was not tracked.

A total of 312 complete screeners were submitted. Of these, 216 (69%) met eligibility criteria and were sent a survey invitation. The most common reason for ineligibility (*n* = 96) was a duplicate screener submission from the same individual (*n* = 66, 69%), followed by not meeting the one-year CHW experience requirement (*n* = 13, 14%), a potentially fraudulent or bot response (*n* = 9), failure to provide contact information (*n* = 5), not identifying as a CHW (*n* = 2), and voluntary withdrawal before survey completion (*n* = 1).

Of the 216 eligible respondents sent a survey invitation, 195 (90%) completed the survey. Sixteen responses were assessed as unusable: eight due to suspicious response patterns ’and eight due to respondents living or working outside the Chicago metropolitan area. This yielded a final analytic sample of 179 usable responses.

Of the 179 respondents, 26 (15%) indicated a preference for phone administration; however, only three completed the survey by phone, as the remaining 23 completed it online before a team member was able to call. Overall, 108 respondents (60%) completed the survey in English and 71 (40%) in Spanish. Among the 176 respondents who completed the survey online, median completion time was 24 min (IQR: 16–33 min). Completion time was not recorded for the three respondents who completed the survey by phone.

### Survey Respondent Characteristics

Demographic and socioeconomic characteristics are presented in Table 2. Respondents were predominantly female (85.5%), Hispanic (63.7%) or non-Hispanic Black (32.4%), and had a mean age of 43.9 years (SD = 11.2). More than half (58.7%) spoke a language other than English at home.

**Table 2:** Demographic and socioeconomic characteristics of chicago-area community health workers (*N* = 179)

Characteristic	*n*	%	Mean (SD)
Age (years)			43.9 (11.2)
*Gender identity*			
Male	21	11.7	
Female	153	85.5	
Non-binary	2	1.1	
*Race/ethnicity*			
Hispanic	114	63.7	
Non-Hispanic Black	58	32.4	
Non-Hispanic Other	7	3.9	
*Speaks a language other than English at home (n = 178)*			
Yes	105	58.7	
No	73	40.8	
*Educational attainment*			
Less than high school through GED/high school diploma	47	26.3	
Some college or associate degree	76	42.5	
Bachelor’s degree or higher	55	30.7	
*Annual household income (n = 178)*			
Less than $15,000	18	10.1	
$15,000–$19,999	6	3.4	
$20,000–$24,999	18	10.1	
$25,000–$34,999	22	12.3	
$35,000–$49,999	51	28.5	
$50,000–$74,999	39	21.8	
$75,000–$99,999	14	7.8	
$100,000 or above	10	5.6	
*Health insurance coverage*			
No current health insurance coverage	50	27.9	
Medicaid, Medical Assistance, CHIP, or children’s state insurance	36	20.1	
Medicare	7	3.9	
Private health insurance (employer, school, or direct purchase)	84	46.9	
Other	10	5.6	
Delayed medical care due to cost in past 12 months	109	61.9	
*Employment status*			
Employed (full or part time)	147	82.1	
Unemployed	18	10.1	
Not in labor force	11	6.2	
*Housing tenure*			
Own	71	55.9	
Rent	100	78.7	
Other arrangement	6	4.7	
Experienced food insecurity in past 12 months	107	59.7	

Percentages may not sum to 100 and rows may not total to 179 due to rounding or missing data. Race/ethnicity was recoded from open-ended responses. NH = Non-Hispanic.

Housing tenure percentages are calculated among respondents who provided a response. Health insurance categories are not mutually exclusive; respondents could select all that apply.

Employment status reflects current status at time of survey; participants were not required to be currently employed to be eligible.

Educational attainment varied across the sample: 26.3% had a high school diploma or less, 42.5% had some college or an associate degree, and 30.7% held a bachelor’s degree or higher. Nearly half of respondents (46.9%) reported private health insurance, while 27.9% reported no current health insurance coverage. Nearly 62% of respondents reported delaying medical care due to cost in the past 12 months. The majority were currently employed (82.1%), and reported renting their home (78.7%). Annual household income was distributed broadly, with the largest proportion (28.5%) reporting incomes between $35,000 and $49,999; 35.9% reported incomes below $25,000. Over half of respondents (59.7%) experienced food insecurity in the past 12 months.

### Health Outcomes and Comparison to Chicago Population Estimates

Health outcomes and behavioral risk factors among CHW respondents, compared to Chicago population estimates from CDC PLACES (2024 release, 2022 BRFSS data) [26], are presented in Table 3.

**Table 3:** Health outcomes and risk behaviors among chicago-area community health workers (*N* = 179) compared to chicago population estimates

	Chicago-area	CHW Sample	Chicago Population^a^				
	*n*	%	Prevalence %	95% CI	z-score	*p*-value^b^	Comparison
*General Health*							
Fair or poor self-rated health	56	31.3	21.1	(19.3, 22.8)	3.34	0.000	Worse
Limited in kind or amount of work due to physical, mental, or emotional problem	62	34.6	–	–			–
*Top 10 Chronic Health Conditions*							
High blood pressure or hypertension	40	22.3	29.7	(27.4, 31.8)	-2.17	0.030	Better
Obesity	36	20.1	31.6	(29.1, 34.0)	-3.31	0.001	Better
Anxiety disorder	34	19.0	–	–			–
Type 2 diabetes	28	15.6	11.9	(10.7, 13.2)	1.53	0.126	NS
Asthma	24	13.4	10.2	(9.1, 11.2)	1.41	0.157	NS
High cholesterol	23	12.8	24.7	(22.2, 27.1)	-3.69	0.000	Better
Depression	19	10.6	12.0	(10.8, 13.1)	-0.58	0.264	NS
Sleep disorder (e.g., insomnia, sleep apnea, narcolepsy, restless legs syndrome)	12	6.7	–	–			–
Chronic musculoskeletal condition (e.g., back pain, osteoarthritis, osteoporosis)	10	5.6	–	–			–
Rheumatologic condition (e.g., rheumatoid arthritis, systemic lupus erythematosus, vasculitis)	9	5.0	–	–			–
*Health Behaviors*							
Adequate fruit consumption (≥5 days/week)	59	33.0	–	–			–
Adequate vegetable consumption (≥5 days/week)	48	26.8	–	–			–
Current tobacco use (cigarette or vape, past 3 months)	15	8.4	11.9	(10.5, 13.1)	-1.45	0.148	NS
Meets physical activity recommendation (≥5 days/week moderate to vigorous activity)	45	25.1	32.6	(29.2, 35.8)	-2.14	0.032	Worse
Inadequate sleep duration (<7 h per night)	88	49.2	37.8	(34.2, 41.4)	3.15	0.002	Worse
Binge drinking in past 3 months (≥4 drinks/day women; ≥5 drinks/day men)	20	11.3	19.0	(16.9, 21.1)	-2.63	0.009	Better
Food insecurity in past 12 months	107	59.7	24.4	(22.5, 26.2)	11	0.000	Worse
*Mental Health*							
Frequent mental distress (≥14 poor mental health days in past 30 days)	42	24.1	16.0	(14.5, 17.5)	2.96	0.003	Worse
Anxiety symptoms (GAD-2 score ≥3)	34	19.1	–	–			–
Depressive symptoms (PHQ-2 score ≥3)	22	12.4	–	–			–
Inadequate emotional support	71	39.7	28.2	(25.6, 31.0)	3.42	0.001	Worse

^a^Chicago population estimates are from CDC PLACES data, 2024 release (2022 BRFSS data). Comparison column reflects direction of difference relative to Chicago population; Chronic health conditions reflect self-reported medical diagnoses; respondents could select all that apply. Conditions without Chicago comparison data (—) were not available in CDC PLACES data.

^b^Indicates *p* < .05 (two-proportion z-test).

#### General Health

Nearly one-third of CHW respondents (31.3%) reported fair or poor general health, a significantly larger proportion than the Chicago population estimate of 21.1% (z = 3.34, *p* < .001). An additional 34.6% reported being limited in the kind or amount of work they could do due to a physical, mental, or emotional problem; no comparable Chicago population estimate was available for this measure.

#### Chronic Health Conditions

**Fig. 1:**
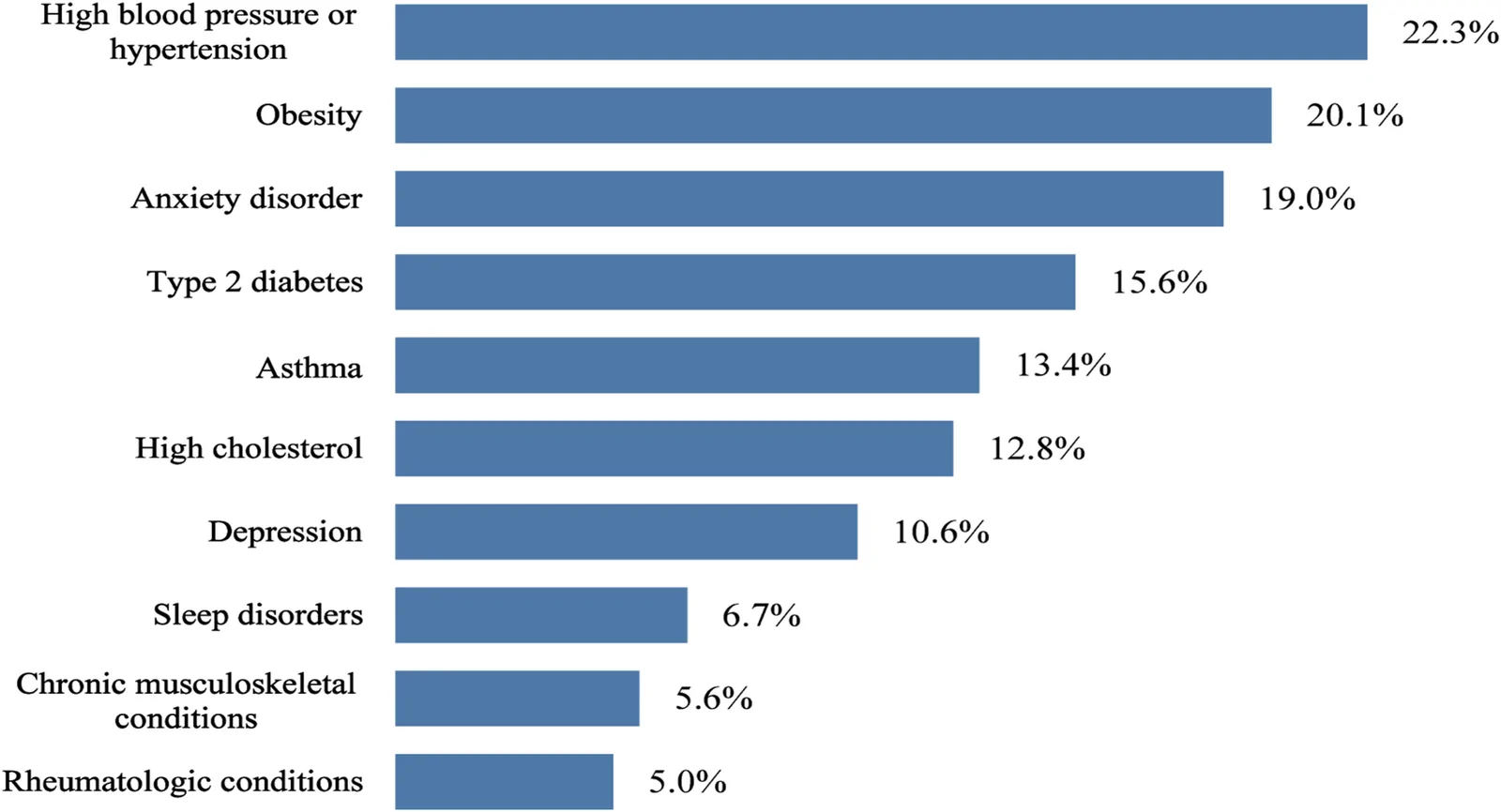
Top 10 reported chronic conditions among CHWs: Chicagoland CHW Survey (*n* = 179)

 The ten most reported chronic conditions among CHWs were high blood pressure or hypertension (22.3%), obesity (20.1%), anxiety disorder (19.0%), type 2 diabetes (15.6%), asthma (13.4%), high cholesterol (12.8%), depression (10.6%), sleep disorders (6.7%), chronic musculoskeletal conditions (5.6%), and rheumatologic conditions (5.0%) (Fig. 1). Compared to Chicago population estimates, CHWs reported significantly lower rates of hypertension (22.3% vs. 29.7%, z = -2.17, *p* = .030), obesity (20.1% vs. 31.6%, z = -3.31, *p* = .001), and high cholesterol (12.8% vs. 24.7%, z = -3.69, *p* < .001). Rates of type 2 diabetes (15.6% vs. 11.9%, z = 1.53, *p* = .126), asthma (13.4% vs. 10.2%, z = 1.41, *p* = .157), and depression (10.6% vs. 12.0%, z = -0.58, *p* = .264) did not differ significantly from Chicago population estimates. Chicago comparison data were not available for anxiety disorder, sleep disorders, musculoskeletal conditions, or rheumatologic conditions.

#### Health Behaviors

One-third of CHWs (33.0%) reported fruit consumption (≥ 5 days/week) and 26.8% reported adequate vegetable consumption (≥ 5 days/week); no Chicago comparison data were available for these measures. CHWs reported significantly higher rates of inadequate sleep compared to Chicago (49.2% vs. 37.8%, z = 3.15, *p* = .002). CHWs reported higher rates of food insecurity than the Chicago population (29.2% vs. 24.4%), but not statistically significantly higher (z = 1.491, *p* = .244). CHWs were significantly less likely to meet physical activity recommendations (25.1% vs. 32.6%, z = -2.14, *p* = .032) and reported significantly lower rates of binge drinking (11.3% vs. 19.0%, z = -2.63, *p* = .009) compared to Chicago population estimates. Current tobacco use (8.4% vs. 11.9%, z = -1.45, *p* = .148) did not differ significantly from the Chicago population.

#### Mental Health

CHWs reported significantly higher rates of frequent mental distress (24.1% vs. 16.0%, z = 2.96, *p* = .003) and inadequate emotional support (39.7% vs. 28.2%, z = 3.42, *p* = .001) compared to Chicago population estimates. Nearly one in five CHWs (19.1%) screened positive for anxiety symptoms (GAD-2 ≥ 3) and 12.4% screened positive for depressive symptoms (PHQ-2 ≥ 3); no Chicago comparison data were available for these screener-based measures.

## Discussion

This pilot study provides one of the first systematic examinations of the health and well-being of CHWs in an urban setting. Among 179 Chicago-area CHWs, we found a mixed health profile. Compared to the general Chicago population, CHWs reported significantly worse outcomes across multiple domains, including general health, mental distress, emotional support, and sleep, yet lower rates of several cardiometabolic conditions. Workforce interventions to address health and well-being have been implemented across the healthcare sector with varying success; most fail to account adequately for differences in healthcare worker types, locations, and tasks [28, 29]. Critically, none have specifically included CHWs, who often have precarious job security, inadequate compensation, and regular exposure to stressful community settings [10]. This study begins to fill that gap.

### Study Feasibility and Community-Engaged Approach

Beyond the health findings, this study demonstrates the feasibility of recruiting and surveying a diverse sample of urban CHWs. 90% of people who were sent the survey completed it with 60% completing in English and 40% in Spanish, reflects both the reach of the recruitment strategy and the relevance of the study to CHWs themselves. The community-engaged approach was central to this success. By involving CHWs and Community Advisory Board members in the design of survey domains, question selection, and recruitment strategies, the study produced an instrument that resonated with CHWs’ lived and work experiences. CHW input led to meaningful modifications to the survey, ensuring that findings reflect priorities identified by the workforce rather than imposed by researchers. As the CHW workforce continues to grow and diversify, community-engaged methods will be essential for generating research that is not only scientifically rigorous but credible and actionable to the workers and communities it aims to serve.

### Health Outcomes

#### Chronic Diseases

Rates of diabetes and asthma among CHWs did not differ significantly from the general Chicago population, a finding that warrants reflection. CHWs are frequently employed in disease management and health education programs focused on exactly these conditions, and it is plausible that CHWs benefit from the same information, skills, and resources they provide to the communities they serve. A similar interpretation may apply to the significantly lower rates of hypertension, obesity, and high cholesterol observed in this sample. It is also possible that these results are due to the “healthy worker effect”, where employed populations appear healthier than the general population due to workforce out-selection [26]. It is also possible that CHWs’ direct engagement in chronic disease prevention and management confers some protective benefit. These two explanations are not mutually exclusive. What is less clear is whether these advantages persist over time. Research has shown that healthcare support workers face elevated cardiovascular and metabolic risks over the course of their careers [8], and longitudinal data will be critical for understanding whether CHW health trajectories diverge from population trends as the demands of the work accumulate. 

#### Mental Health and Emotional Burden

This sample of CHWs reported significantly higher rates of frequent mental distress and inadequate emotional support compared to the Chicago population. Nearly one in five screened positive for anxiety symptoms and one in eight for depressive symptoms, suggesting substantial mental health needs. These findings are consistent with literature on compassion fatigue, secondary traumatic stress, and moral injury among frontline workers serving individuals experiencing poverty, violence, and chronic illness [6, 29]. Research has specifically identified the need for workplace supports to reduce vicarious trauma and burnout among CHWs, supports that remain inconsistently available.

Nearly 40% of CHWs reported inadequate emotional support, a notable finding for a workforce whose core function is providing that support to others. This pattern of giving without receiving support is a recognized precursor to burnout in care-based professions [30]. For a workforce that is predominantly women of color, culturally specific pressures may compound these occupational stressors. Future analyses using the Superwoman Schema Scale [23] will examine how expectations of strength and self-reliance shape CHW health and coping.

#### Economic Hardship, Sleep, and Physical Activity

Although food insecurity among CHWs (29.2%) did not differ significantly from the Chicago population estimate (24.4%), the burden itself is notable. Combined with high rates of delayed medical care due to cost (61.9%), and 23.6% of respondents earning below $25,000 annually, these findings point to meaningful economic precarity. This raises important questions about the sustainability of this workforce model without deliberate economic investment.

CHWs were also significantly more likely to report inadequate sleep (49.2% vs. 37.8%) and less likely to meet physical activity recommendations compared to Chicago adults. Lower rates of leisure-time physical activity may reflect time constraints and fatigue rather than lack of motivation, a distinction that matters for how employers design wellness programming.

### Implications for Employers and Policymakers

A workforce navigating their own food insecurity, delayed medical care, and significant mental health burden cannot sustain demanding community health work without targeted support. Employers should consider building living wages, subsidized healthcare, paid sick leave, and structured mental health support into CHW employment benefits. Workload and scheduling practices that drive sleep deprivation deserve attention as occupational health concerns rather than individual lifestyle issues.

At the policy level, most states are currently developing CHW credentialing and reimbursement structures [7]. Embedding workforce health protections into these frameworks would ensure policy decisions are informed by CHW experiences. Healthcare systems integrating CHWs into care teams should ensure hiring, supervision, and job support structures account for the unique demands of bridging clinical and community environments [31, 32]. Investment in CHW workforce health is likely to yield returns in reduced turnover and more effective service delivery in communities with the greatest need.

### Limitations

This pilot study has several limitations. First, the convenience sample limits generalizability. While the predominantly female and Hispanic composition of this sample reflects this region’s CHW workforce it does limit broader comparability to Chicago’s population. Second, inherent to surveys, there is potential bias to our use of self-reported outcomes. Third, this study’s cross-sectional design precludes our ability to evaluate the impact of the bias involving the healthy worker effect (where less healthy members of the sample aren’t identified). Finally, population comparisons should be interpreted cautiously given two-to-three-year gap between the CHW survey (2026) and Chicago comparison data (2022) [26].

This pilot study has several notable strengths. It provides one of the first systematic examinations of CHW health in an urban U.S. setting, addressing a significant gap in the occupational health literature. The community-engaged design strengthens the relevance and credibility of the findings. The bilingual survey design and high completion rate reflect both the accessibility of the study and its resonance with the CHW workforce. The use of validated, standardized measures across multiple health domains enables meaningful comparisons to population-level data and supports future replication.

## Conclusion

This study documents a CHW workforce carrying substantial physical and mental health burden and economic hardship while playing an essential role in connecting underserved communities to care. The findings challenge the assumption that good CHW health can be inferred from employment status or proximity to health systems. As the CHW workforce grows and becomes more formally integrated into healthcare delivery, protecting CHW health is not a peripheral concern but a prerequisite for a sustainable workforce. Phase 2 interviews and Phase 3 longitudinal follow-up will deepen and extend these findings and will equip employers, health systems, and policymakers with the necessary evidence to build workplaces that protect the workers who protect their communities.

## Data Availability

The data that support the findings of this study are not publicly available due to participant confidentiality but are available from the corresponding author upon reasonable request.

## Declarations

### Disclaimer

The views and conclusions contained in this document are those of the authors and should not be interpreted as representing the official policies, either expressed or implied, of the NIH.

## References

[1] American Public Health Association Community Health Worker Section (n.d.). Community health workers. https://www.apha.org/apha-communities/member-sections/community-health-workers 10.2190/NS.20.3.l 20943481

[2] Rothschild, S. K., Martin, M. A., Swider, S. M., et al. (2014). Mexican American trial of community health workers: A randomized controlled trial of a community health worker intervention for Mexican Americans with type 2 diabetes mellitus. *American Journal of Public Health*, *104*(8), 1540–1548. 10.2105/ajph.2013.301439 PMC4103223 23947316

[3] Krieger, J., Song, L., & Philby, M. (2015). Community health worker home visits for adults with uncontrolled asthma: The HomeBASE Trial randomized clinical trial. *JAMA Internal Medicine*, *175*(1), 109–117. 10.1001/jamainternmed.2014.6353 25419871

[4] Wells, K. J., Luque, J. S., Miladinovic, B., et al. (2011). Do community health worker interventions improve rates of screening mammography in the United States? A systematic review. *Cancer Epidemiology Biomarkers & Prevention*, *20*(8), 1580–1598. 10.1158/1055-9965.EPI-11-0276 PMC3153589 21653645

[5] Kangovi, S., Mitra, N., Grande, D., et al. (2014). Patient-centered community health worker intervention to improve posthospital outcomes: A randomized clinical trial. *JAMA Internal Medicine*, *174*(4), 535–543. 10.1001/jamainternmed.2013.14327 24515422

[6] Ignoffo, S., Margellos-Anast, H., Banks, M., Morris, R., & Jay, K. (2022). Clinical integration of community health workers to reduce health inequities in overburdened and under-resourced populations. *Population Health Management*, *25*(2), 280–283. 10.1089/pop.2021.0376 PMC9058862 35442790

[7] Association of State and Territorial Health Officials (n.d.). Community health workers. https://www.astho.org/topic/population-health-prevention/healthcare-access/community-health-workers/

[8] Alhajaji, R., Alfahmi, M. Z., Alshaikhi, S. A., et al. (2025). The influence of workplace stressors on the risk of cardiovascular diseases among healthcare providers: A systematic review. *Frontiers in Psychiatry*, *16*, 1461698. 10.3389/fpsyt.2025.1461698 PMC12409286 40917244

[9] Nigam, J. A., Barker, R. M., Cunningham, T. R., Swanson, N. G., & Chosewood, L. C. (2023). Vital signs: Health worker–perceived working conditions and symptoms of poor mental health — Quality of Worklife Survey, United States, 2018–2022. *Morbidity and Mortality Weekly Report*, *72*, 1197–1205. 10.15585/mmwr.mm7244e1 PMC10629752 37917563

[10] Rosenthal, E., Menkin, P., & St. John, J. (2018). The community health worker core consensus (C3) project: A report of the C3 project phase 1 and 2, together leaning toward the sky. Texas Tech University Health Sciences Center. https://0d6c00fe-eae1-492b-8e7d-80acecb5a3c8.filesusr.com/ugd/7ec423_2b0893bcc93a422396c744be8c1d54d1.pdf

[11] Bureau of Labor Statistics (2024). Occupational employment and wages, May 2024: 21-1094 community health workers. U.S. Department of Labor. https://www.bls.gov/oes/current/oes211094.htm

[12] Kollar, M., & Scherer, Z. (2025). *Income in the United States: 2024 (Current Population Reports, P60-286)*. U.S. Census Bureau, U.S. Government Publishing Office.

[13] Sabo, S., Allen, C. G., Sutkowi, K., & Wennerstrom, A. (2017). Community health workers in the United States: Challenges in identifying, surveying, and supporting the workforce. *American Journal of Public Health*, *107*(12), 1964–1969. 10.2105/ajph.2017.304096 PMC5678391 29048953

[14] Rosenthal, E. L. (1998). The final report of the national community health advisor study. https://crh.arizona.edu/sites/default/files/2022-04/CAHsummaryALL.pdf

[15] Chari, R., Chang, C. C., Sauter, S. L., Sayers, P., Huang, E. L., W., & Fisher, G. G. (2024). *NIOSH worker well-being questionnaire (WellBQ) (DHHS [NIOSH] Publication No. 2021 – 110, rev. ed)*. National Institute for Occupational Safety and Health, Centers for Disease Control and Prevention. 10.26616/NIOSHPUB2021110revised052024

[16] Centers for Disease Control and Prevention. (2022). *Behavioral risk factor surveillance system survey questionnaire*. U.S. Department of Health and Human Services.

[17] Sallis, J. F., Pinski, R. B., Grossman, R. M., Patterson, T. L., & Nader, P. R. (1988). The development of self-efficacy scales for health-related diet and exercise behaviors. *Health Education Research*, *3*(3), 283–292. 10.1093/her/3.3.283

[18] McNeely, J., Wu, L., Subramaniam, G., et al. (2016). Performance of the Tobacco, Alcohol, Prescription Medication, and Other Substance Use (TAPS) Tool for substance use screening in primary care patients. *Annals of Internal Medicine*, *165*, 690–699. 10.7326/M16-0317 PMC5291717 27595276

[19] Arroll, B., Goodyear-Smith, F., Crengle, S., et al. (2010). Validation of PHQ-2 and PHQ-9 to screen for major depression in the primary care population. *Annals of Family Medicine*, *8*(4), 348–353. 10.1370/afm.1139 PMC2906530 20644190

[20] Plummer, F., Manea, L., Trepel, D., & McMillan, D. (2016). Screening for anxiety disorders with the GAD-7 and GAD-2: A systematic review and diagnostic meta-analysis. *General Hospital Psychiatry*, *39*, 24–31. 10.1016/j.genhosppsych.2015.11.005 26719105

[21] Hughes, M. E., Waite, L. J., Hawkley, L. C., & Cacioppo, J. T. (2004). A short scale for measuring loneliness in large surveys: Results from two population-based studies. *Research on Aging*, *26*(6), 655–672. 10.1177/0164027504268574 PMC2394670 18504506

[22] Williamson, A. L. C., Stickley, M. M., Armstrong, T. W., Jackson, K., & Console, K. (2022). Diagnostic accuracy of the Primary Care PTSD Screen for DSM-5 (PC-PTSD-5) within a civilian primary care sample. *Journal of Clinical Psychology*, *78*(11), 2299–2308. 10.1002/jclp.23405 35763419

[23] Woods-Giscombé, C. L. (2010). Superwoman schema: African American women’s views on stress, strength, and health. *Qualitative Health Research*, *20*(5), 668–683. 10.1177/1049732310361892 PMC3072704 20154298

[24] Linzer, M., McLoughlin, C., Poplau, S., Goelz, E., Brown, R., & Sinsky, C. (2022). The Mini Z worklife and burnout reduction instrument: Psychometrics and clinical implications. *Journal of General Internal Medicine*. 10.1007/s11606-021-07278-3 PMC9411313 35048290

[25] Harris, P. A., Taylor, R., Thielke, R., Payne, J., Gonzalez, N., & Conde, J. G. (2009). Research electronic data capture (REDCap): A metadata-driven methodology and workflow process for providing translational research informatics support. *Journal of Biomedical Informatics*, *42*(2), 377–381. 10.1016/j.jbi.2008.08.010 PMC2700030 18929686

[26] Centers for Disease Control and Prevention (2026, April 5). PLACES. https://www.cdc.gov/places

[27] StataCorp. (2023). *Stata statistical software: Release 18*. StataCorp LLC.

[28] Brand, S. L., Coon, J. T., Fleming, L. E., Carroll, L., Bethel, A., & Wyatt, K. (2017). Whole-system approaches to improving the health and wellbeing of healthcare workers: A systematic review. *PLOS ONE*, *12*, e0188418. 10.1371/journal.pone.0188418 PMC5714334 29200422

[29] Chan, C. W., & Perry, L. (2012). Lifestyle health promotion interventions for the nursing workforce: A systematic review. *Journal of Clinical Nursing*, *21*, 2247–2261. 10.1111/j.1365-2702.2012.04213.x 22788559

[30] Achore, M., Hackett, M., & Ramirez, T. (2026). Preventing burnout: Community health worker perspectives on professional development and emotional labor overload — A qualitative descriptive study. *PLOS ONE*, *21*(4). 10.1371/journal.pone.0346430. Article e0346430. PMC13046155 41926356

[31] Witmer, A., Seifer, S. D., Finocchio, L., Leslie, J., & O’Neil, E. H. (1995). Community health workers: Integral members of the health care work force. *American Journal of Public Health*, *85*(8 Pt 1), 1055–1058. 10.2105/AJPH.85.8_Pt_1.1055 PMC1615805 7625495

[32] Perez, L. M., & Martinez, J. (2008). Community health workers: Social justice and policy advocates for community health and well-being. *American Journal of Public Health*, *98*(1), 11–14. 10.2105/AJPH.2006.100842 PMC2156047 18048789

